# Heat Shock Protein Inhibitors Show Synergistic Antibacterial Effects with Photodynamic Therapy on Caries-Related Streptococci *In Vitro* and *In Vivo*

**DOI:** 10.1128/msphere.00679-22

**Published:** 2023-02-28

**Authors:** Zichen Zhang, Yaoting Ji, Danfeng Liu, Shuhui Zhou, Zijun Wang, Rourong Chen, Ting Li, Boxuan Zhao, Hantao Yao, Minquan Du

**Affiliations:** a The State Key Laboratory Breeding Base of Basic Science of Stomatology (Hubei-MOST) and Key Laboratory of Oral Biomedicine Engineering Ministry of Education, School and Hospital of Stomatology, Wuhan University, Wuhan, China; Antimicrobial Development Specialists, LLC

**Keywords:** caries, chlorin e6, heat shock protein inhibitors, photodynamic therapy, *Streptococcus*

## Abstract

Caries are chronic infections in which the cariogenic biofilm plays a critical role in disease occurrence and progression. Photodynamic therapy (PDT) is a new effective treatment that is receiving wide attention in the antibacterial field, but it can lead to the upregulation of heat shock proteins (HSPs), which enhances bacterial resistance. Herein, we incorporated HSP inhibitors with PDT to evaluate the effect on Streptococcus mutans, Streptococcus sobrinus, and Streptococcus sanguinis under planktonic conditions and on cariogenic biofilms. Additionally, a model of caries was established in 2-week-old rats, and anticaries properties were evaluated by Keyes’ scoring. Importantly, the combination of HSP inhibitors and PDT had outstanding efficiency in inhibiting the growth of tested Streptococcus strains and the formation of either monomicrobial or multispecies biofilms *in vitro*. In addition, the quantity of colonized streptococci and the severity of carious lesions were also distinctly suppressed in vivo. Overall, the synergistic application of HSP inhibitors and PDT has promising potential in the prevention and treatment of dental caries.

**IMPORTANCE** Effective therapies for the prevention and control of caries are urgently needed. Cariogenic streptococci play a key role in the occurrence and progression of caries. Recently, photodynamic therapy has been demonstrated to have good antibacterial efficiency, but it can cause a heat shock response in bacteria, which may weaken its practical effects. We indicate here an effective therapeutic strategy of combining heat shock protein inhibitors and photodynamic therapy, which shows excellent inhibition toward three dominant streptococci related to caries and suppression of carious progression in a rat model. Further development for clinical application is promising.

## INTRODUCTION

Caries is one of the most common prevalent oral diseases worldwide (1–3) and is one of the main drivers of tooth loss in addition to periodontal diseases (4). As a major health burden, caries has affected the majority of people from all age groups in most countries, and its prevalence rate has reached 34.1% in 2015 worldwide (5, 6). As a chronic infectious disease, the occurrence of caries begins with a deleterious shift of the microbiota in oral biofilms, where the Gram-positive oral streptococci have a higher cell density. Among those streptococci, Streptococcus mutans strongly correlates with caries progression because of its robust acidogenicity, aciduricity, and ability to assemble insoluble exopolysaccharide-rich matrix (7–9). Based on the results of previous studies, the proportion of S. mutans is less than 2% in initial oral biofilm, while it can reach 10% in early enamel lesions (10, 11). Additionally, in dentine lesions, S. mutans accounts for about 30% of the total microbiota (12). Other oral streptococci also constitute an important arm in biofilm formation and cariogenicity. For instance, Streptococcus sanguinis colonizes on the tooth surface as one of the major pioneer bacteria (13, 14), while Streptococcus sobrinus has a higher rate of acid production than S. mutans in a low-pH environment (15). When it is frequently exposed to fermentable carbohydrates, excessive organic acid produced by bacteria in biofilms results in a repeated low-pH microenvironment, which leads to sustained demineralization in dental hard tissue, and tooth decay occurs. Hence, arresting colonization and biofilm formation of cariogenic bacteria could be a crucial therapeutic target of dental caries.

Recently, photodynamic therapy (PDT) has been brought into focus as an effective antibacterial method that functions by generating singlet oxygen (^1^O_2_) and reactive oxygen species (ROS) to destroy important intracellular or extracellular biomolecules when photosensitizers (PSs) are illuminated with light of a specific wavelength in an environment containing oxygen (16–18). As an essential part of PDT, PSs have been developed for two generations, and the second generation has been widely investigated in various domains. Among the second-generation PSs, chlorin e6 (Ce6) is a natural PS derived from chlorophyll, which has superior properties, such as good absorption of red light, a short photosensitizing period, and minimal side effects (19, 20). Currently, PDT has already been used in the clinic because of its high selectivity without genotoxicity, invasiveness, or mutagenic effects (21, 22). However, PDT still has several limitations, and one is that it may upregulate the expression of heat shock proteins (HSPs) in cells that raise their resistance to PDT (23–25). HSPs are among the most highly conserved chaperones and exhibit homology in eukaryotes and prokaryotes (26, 27). HSPs are indispensable for fundamental functions, such as promoting correct assembly of unfolded polypeptides and degrading abnormal proteins (28, 29), while playing instrumental roles in cell adaptation to environmental stress, including oxidative stress and acidic or alkaline pH (30), achieving protective functions in cells (31, 32). There are two major HSP families in eukaryotes that are associated with protecting cells from adverse environmental stresses: the GroEL family and DnaK family, corresponding to HSP 60 and HSP 70 in prokaryotes, respectively (31, 33). Benefiting from their protective abilities under extreme conditions, HSPs may discount the therapeutic efficiency of PDT and even lead to the survival of residual bacteria after treatment. Therefore, it is a feasible assumption that HSP inhibitors can be added during PDT to minimize this inefficiency. This assumption has currently been demonstrated in several cancer-related studies (34, 35); however, no evidence on the combined administration of PDT and HSP inhibitors exists in the context of the antibacterial field.

The objective of this study was to explore the inhibitory effect of PDT combined with HSP inhibitors on cariogenic bacteria under planktonic conditions and in biofilms and verify the effect on the occurrence and progression of caries lesions in a rat model to find a new strategy for the control and prevention of caries.

## RESULTS

### The antibacterial effect of Ce6-PDT on S. mutans, S. sobrinus, and S. sanguinis.

**FIG 1:**
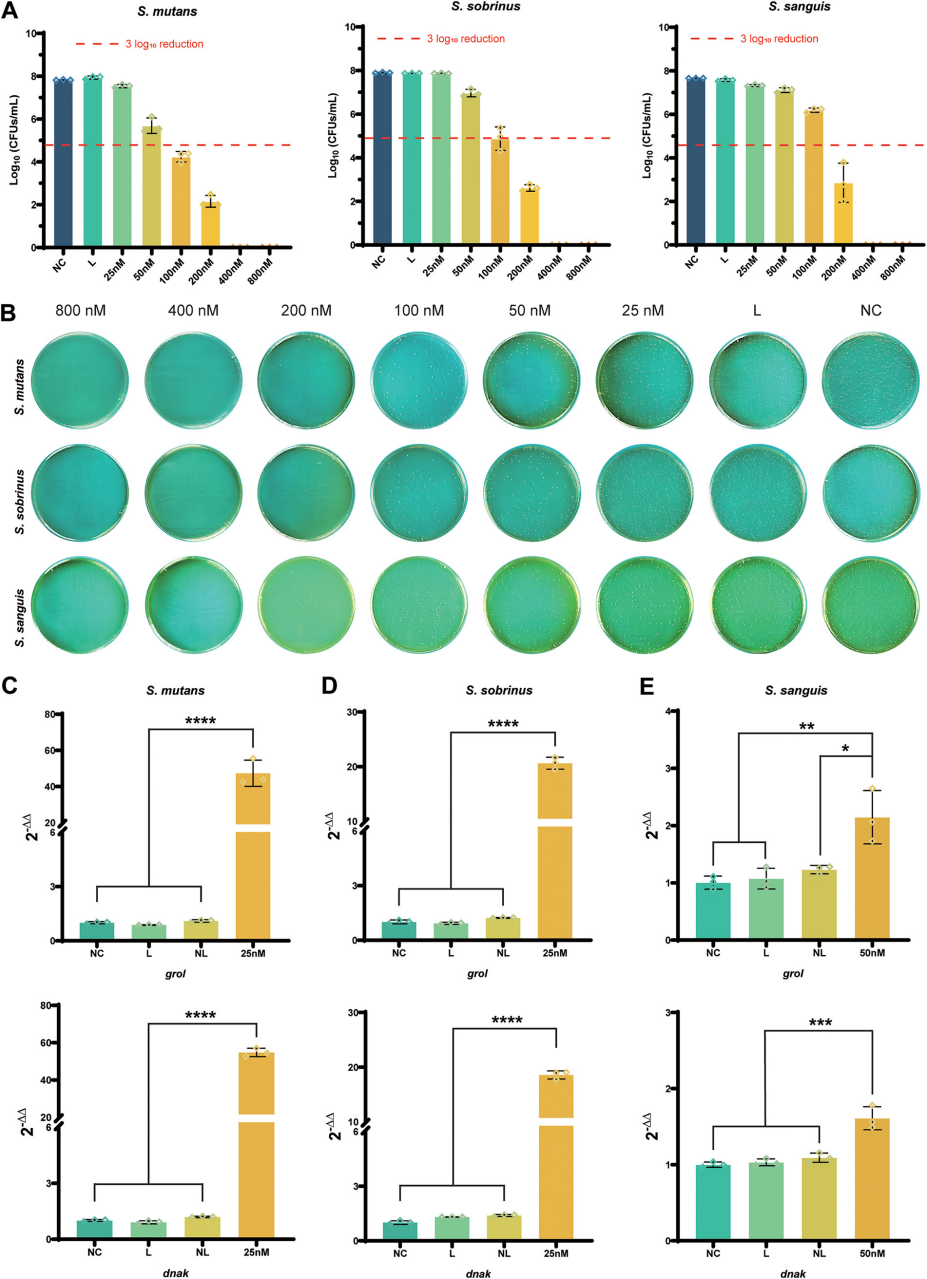
Antibacterial effects of PDT at different concentrations of Ce6 on S. mutans, S. sanguinis, and *S. sobrinus*. (A) The lethal doses of Ce6-PDT toward three bacteria strains were detected by CFU counting. (B) Representative agar plates are displayed. (C to E) Expression levels of *grol* and *dnak* genes are normalized by the cycling threshold (2^−ΔΔ^*^CT^*) method relative to the corresponding 16S rRNA gene in S. mutans (C), *S. sobrinus* (D), and *S. sanguis* (E); L, received irradiation only; NL, received Ce6 without irradiation; NC, received no interventions. Results are shown as mean ± SD (*n* = 3); *, *P < *0.05; **, *P < *0.01; ***, *P < *0.001; ****, *P < *0.0001.

Bacterial quantities were determined by spread plate technique and CFU counting. As shown in Fig. 1A and B, Ce6 exhibited complete bactericidal properties toward three bacterial strains at concentrations of 400 nM and 800 nM. At a concentration of 200 nM, decreases in *S. sobrinus* and S. sanguinis were approximately 5 log_10_, and the decrease even reached nearly 6 log_10_ in S. mutans, compared with the normal control group. In the group treated with 100 nM Ce6, decreases of S. mutans and *S. sobrinus* were 4 log_10_ and 3 log_10_, respectively. Additionally, the reductions of three bacteria were lower than 2 log_10_ when the concentration of Ce6 was below 50 nM. According to the definition mentioned in the Methods, the lethal dose (LD) of Ce6 on S. mutans and *S. sobrinus* was 100 nM and was 200 nM on S. sanguinis.

### Ce6-PDT upregulates the expression of *grol* and *dnak* genes.

To test the potential possibility of whether Ce6-PDT had effects on the expression of HSPs, we performed quantitative real-time PCR (qRT-PCR) to detect mRNA levels of *grol* and *dnak* genes, and 1/4 LD Ce6 was used in this experiment. Our data indicated that gene expression exhibited significant upregulation after PDT. Specifically, in S. mutans (Fig. 1C), the expression of *grol* was upregulated nearly 50-fold, and the upregulation of *dnak* was about 60-fold compared with the negative-control (NC) group. As for *S. sobrinus* (Fig. 1D), the expression of *grol* and *dnak* was upregulated approximately 20-fold. However, the mRNA level of *grol* in S. sanguinis was only twice as much as in the NC group (Fig. 1E), while the change in *dnak* expression was less than 2-fold. For all three strains, treatment with light or Ce6 alone did not impact the mRNA expression of the two target genes compared to the NC group. These results demonstrate that Ce6-PDT has the ability to upregulate the expression of HSP-encoding genes.

### Synergistic inhibitory effect of Ce6-PDT and HSP inhibitors on planktonic bacteria.

**FIG 2:**
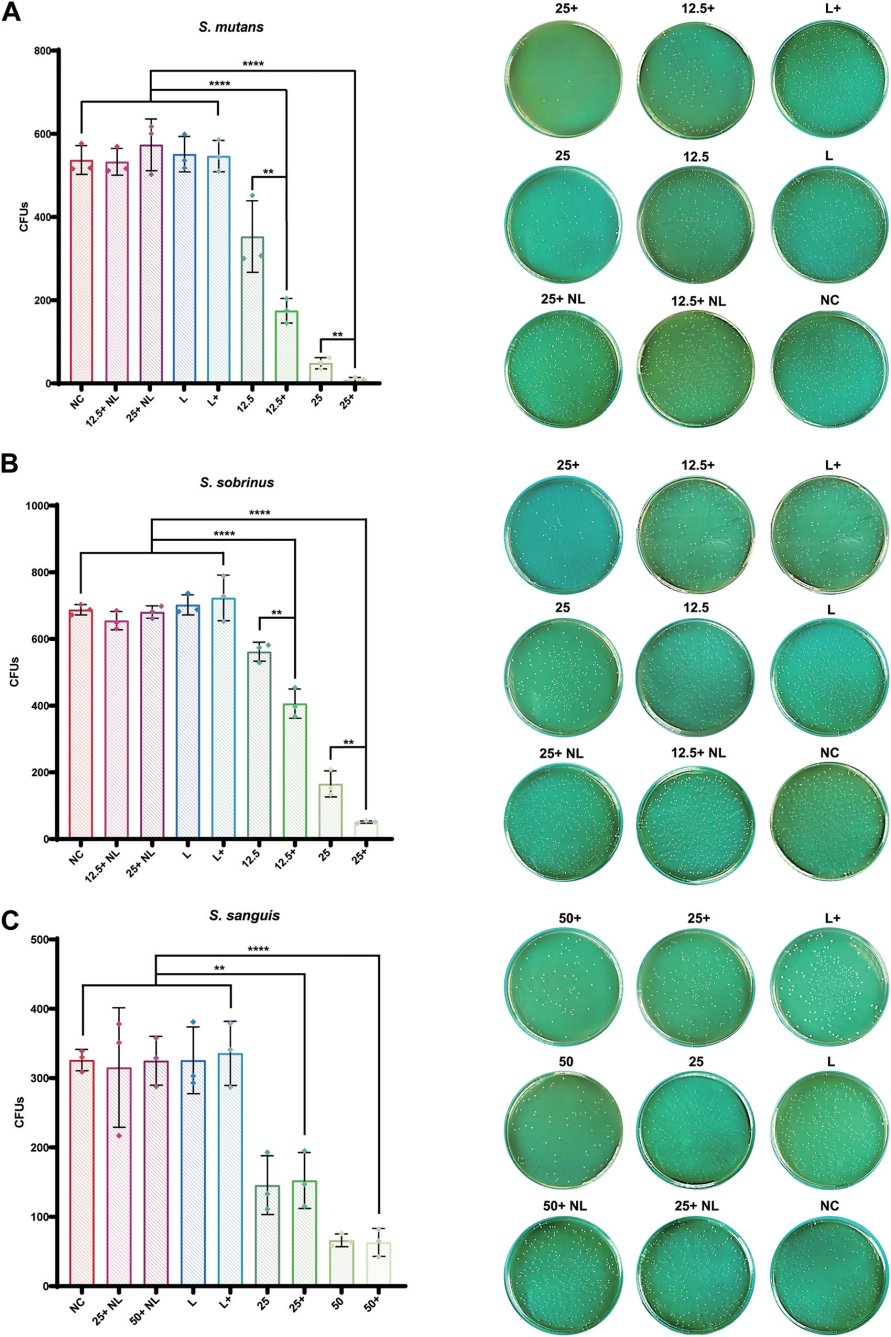
(A to C) Antibacterial effects of Ce6-PDT combined with HSP inhibitors on S. mutans (A), *S. sobrinus* (B), and S. sanguinis (C); 12.5+, 25+, 50+, received PDT with HSP inhibitors at different concentrations (nM) of Ce6; 12.5, 25, 50, received PDT at different concentrations of Ce6 without HSP inhibitors; 12.5+NL, 25+NL, 50+NL, received Ce6 and HSP inhibitors without irradiation; L+, received HSP inhibitors after irradiation; L, received irradiation only; NC, received no interventions. The results are shown as mean ± SD (*n* = 3); **, *P < *0.01; ****, *P < *0.0001.

As shown by our data (Fig. 2), the proliferation of planktonic bacteria could not be significantly inhibited under the treatment of single light (L group), drug without light (NL group), or inhibitors with light (L+ group). In S. mutans (Fig. 2A), antibacterial effects were significantly enhanced, and there were fewer residual bacteria on agar plates in the presence of inhibitors than in the corresponding PDT (12.5 and 25 nM Ce6) group (*P* < 0.005). Additionally, a similar trend was observed in *S. sobrinus* (Fig. 2B). However, no significant difference was found between the PDT (25 and 50 nM Ce6) group and the PDT with inhibitors (Ce6+) group in S. sanguinis (Fig. 2C). These findings corroborate our gene expression results.

### Synergistic inhibitory effect of Ce6-PDT and HSP inhibitors on biofilm formation.

**FIG 3:**
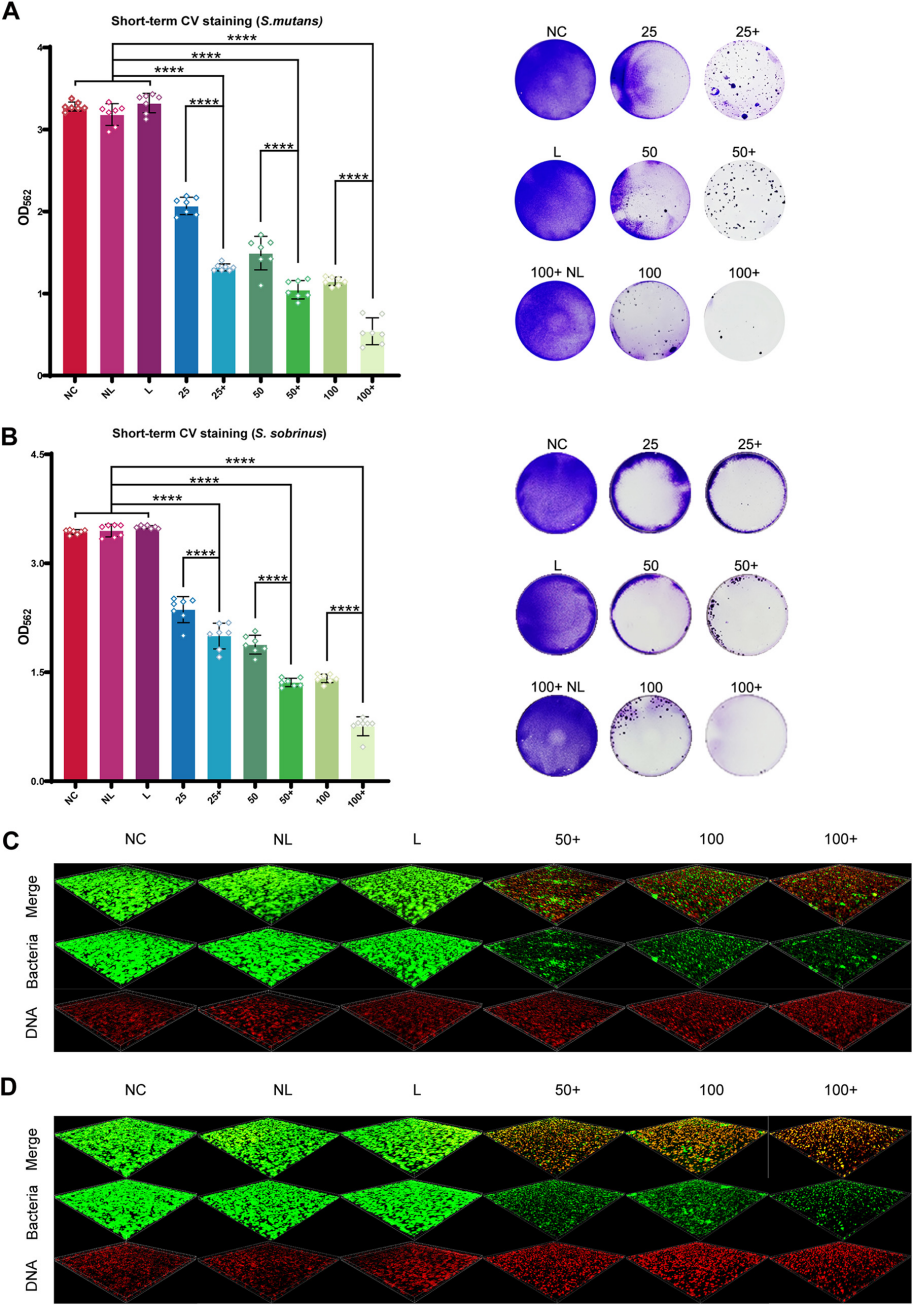
Inhibitory effects of Ce6-PDT combined with HSP inhibitors on monomicrobial biofilm. (A and B) Total biomasses of biofilms are presented by the absorbance of crystal violet in S. mutans (A) and *S. sobrinus* (B). Representative photographs of stained biofilms are displayed on the right. The results are shown as mean ± SD (*n* = 7); ****, *P < *0.0001. (C) Live/dead bacterial staining viewed by CLSM. Red fluorescence indicates dead bacteria, and green fluorescence indicates live bacteria; 25+, 50+, 100+, received PDT with HSP inhibitors at different concentrations (nM) of Ce6; 25, 50, 100, received PDT at different concentrations of Ce6 without HSP inhibitors; 100+NL, received 100 nM Ce6 and HSP inhibitors without irradiation; L, received irradiation only; NC, received no interventions.

Synergistic effects on the inhibition of short-term monomicrobial biofilm formation were extremely obvious in both S. mutans (Fig. 3A) and *S. sobrinus* (Fig. 3B), as evidenced by crystal violet (CV) staining. Specifically, the total biomass of biofilms showed a decrease after being lighted with Ce6 and inhibitors in a dose-dependent manner, and there was almost no biofilm formation in the 100 nM Ce6+ group. Additionally, biofilm biomass was significantly reduced in Ce6+ groups compared to in corresponding Ce6 groups (*P < *0.0001). To our surprise, PDT using Ce6 at a lower dose could reach a similar inhibitory efficiency on biofilms in the presence of inhibitors as observed in groups using Ce6 at higher doses. After staining with a bacterial viability kit, live cells were marked as green, and dead cells were marked as red based on the different penetrating ability of the two fluorescence stains. A consistent result was observed by confocal laser scanning microscopy (CLSM) where both formed monomicrobial biofilms had lower quantities of living bacteria (green fluorescence) in Ce6+ groups than in Ce6 groups (Fig. 3C and D).

**FIG 4:**
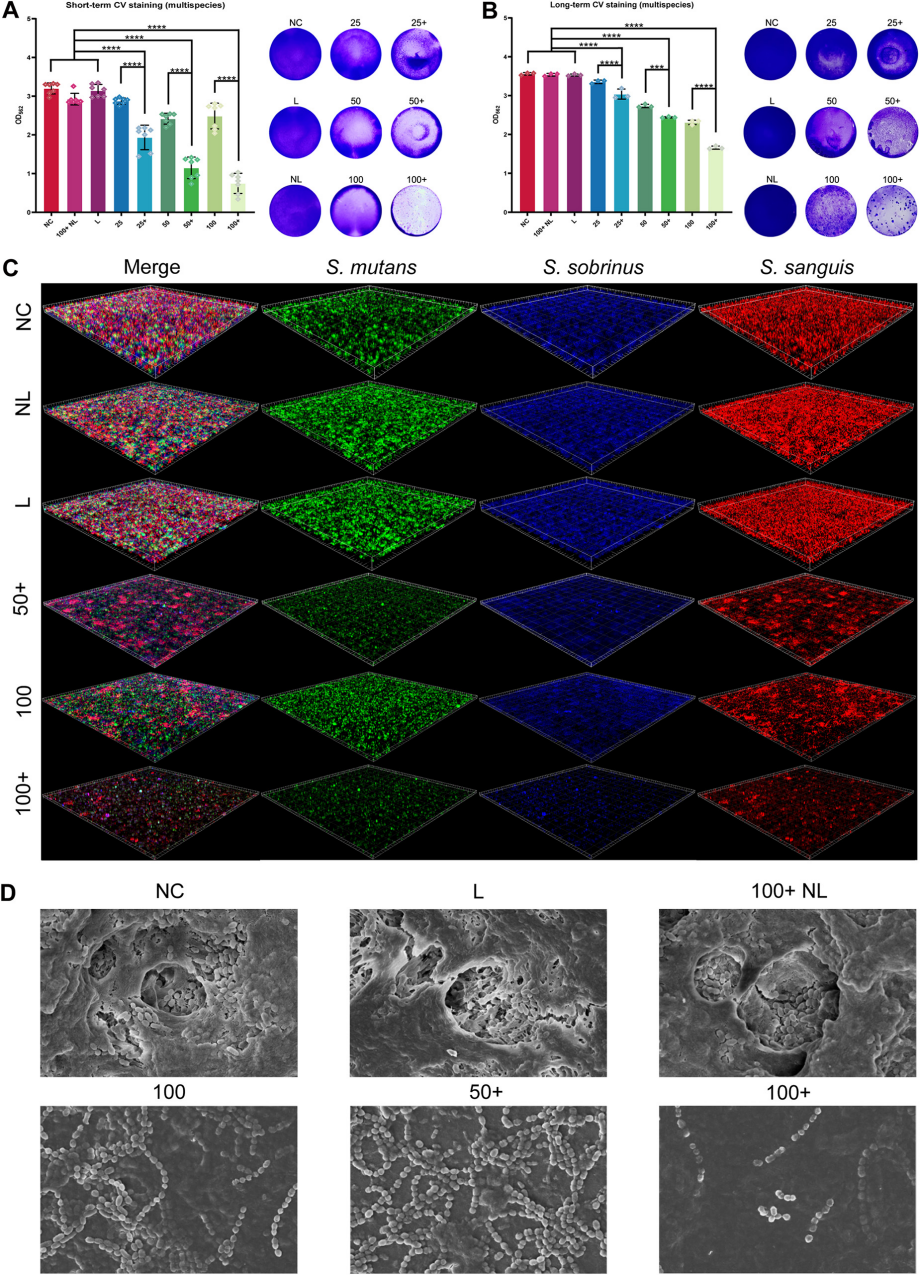
Inhibitory effects of Ce6-PDT combined with HSP inhibitors on multispecies biofilms. (A and B) Total biomasses of multispecies biofilms are presented by the absorbance of crystal violet after short-term (A) and long-term (B) treatment. Representative photographs of stained biofilms are displayed on the right. The results are shown as mean ± SD (*n* = 7); ***, *P < *0.001; ****, *P < *0.0001. (C) FISH in short-term biofilms viewed by CLSM; green fluorescence labeled S. mutans, blue fluorescence labeled *S. sobrinus*, and red fluorescence labeled S. sanguinis. (D) The population and structure of long-term biofilm viewed by SEM at a magnification of ×5,000; 25+, 50+, 100+, received PDT with HSP inhibitors at different concentrations (nM) of Ce6; 25, 50, 100, received PDT at different concentrations of Ce6 without HSP inhibitors; 100+NL, received 100 nM Ce6 and HSP inhibitors without irradiation; L, received irradiation only; NC, received no interventions.

The synergistic inhibitory effect on short-term multispecies biofilm formation had the same trend as the monomicrobial biofilm. Biofilm biomass decreased sharply in the presence of inhibitors during PDT (Fig. 4A). Additionally, the use of inhibitors in PDT with Ce6 at lower concentrations had a better effect on inhibition of biofilm formation than PDT with Ce6 at higher concentrations. As shown by fluorescence *in situ* hybridization (FISH) (Fig. 4C) in which tested bacteria were labeled with single-stranded nucleic acid probes to show corresponding fluorescence, quantities of S. mutans (green fluorescence), *S. sobrinus* (blue fluorescence), and S. sanguinis (red fluorescence) were obviously decreased, and the thickness of multispecies biofilm was thinner in the Ce6+ group than in the corresponding Ce6 group.

The synergistic inhibitory effect on biofilm formation was also significant in long-term multispecies biofilm formation (Fig. 4B). When biofilms were observed by field-emission scanning electron microscopy (FE-SEM), we found that the extracellular matrix network of biofilms was successfully constructed in control groups, while biofilm structures were formed incompletely under PDT (Fig. 4D). Based on the destruction of biofilm structure, the added inhibitors further reduced bacterial aggregation, as shown by the decrease in cell binding.

### *In vivo* experiment.

**FIG 5:**
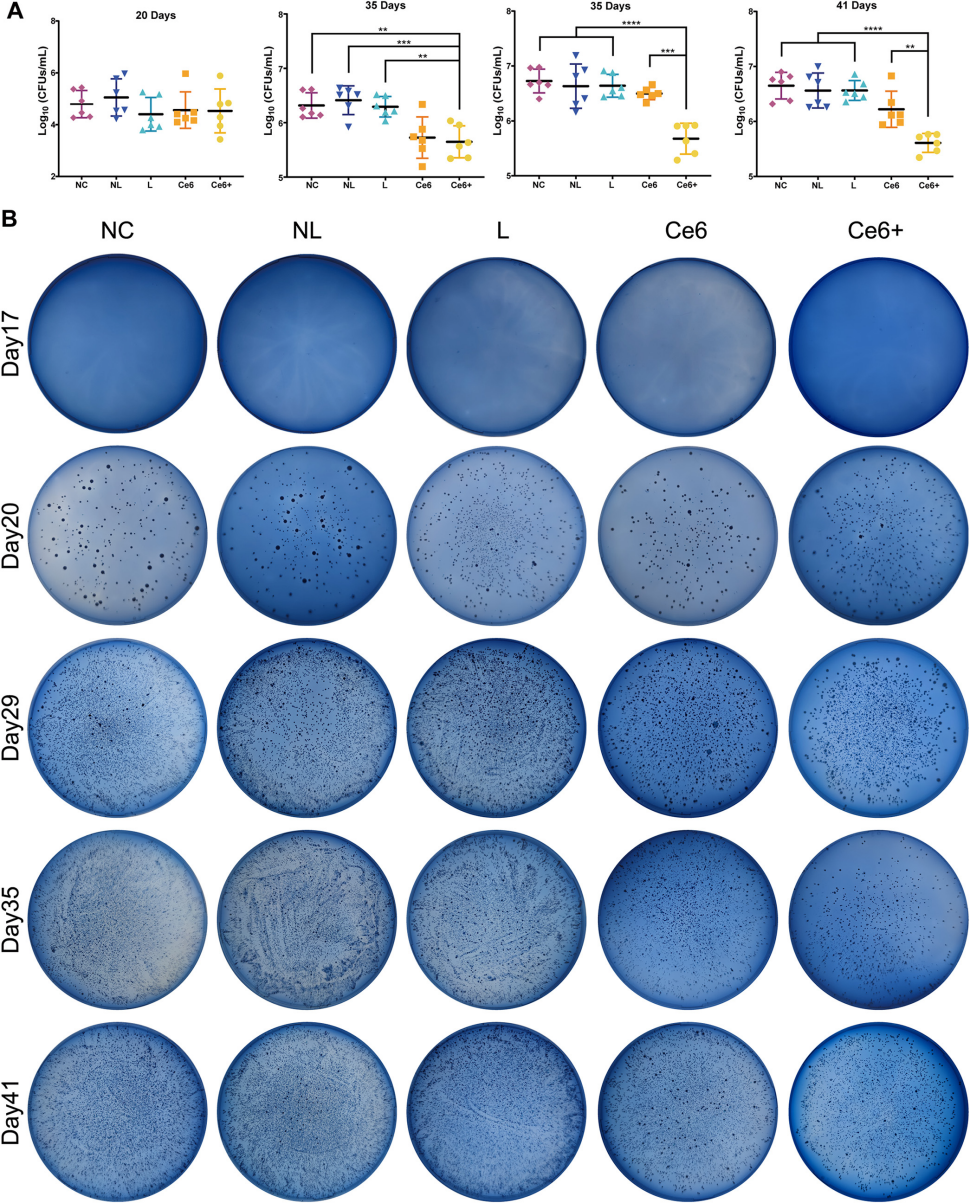
Antibacterial effects of Ce6-PDT combined with HSP inhibitors *in vivo*. (A) CFU counting for streptococci collected from the occlusal surface of rat molars. The results are shown as mean ± SD (*n* = 6); **, *P < *0.01; ***, *P < *0.001; ****, *P < *0.0001. (B) Representative photographs of each group are displayed.

Subsequently, we focused our experiments to further assess the synergistic effect of Ce6-PDT and HSP inhibitors in a rat model. First, antibacterial properties were verified *in vivo* via the plate-spread technique (Fig. 5B) and CFU counting (Fig. 5A). The selective Mitis Salivarius agar (MSA) plates, used in the present study to examine the bacterial amount on the tooth surface, only permit the growth of streptococci species. Multiple bacterial suspensions consisting of S. mutans, *S. sobrinus*, and S. sanguinis were inoculated successfully in the NC, NL, L, Ce6, and Ce6+ groups on the 20th day, and there was almost no difference among these groups. On the 29th day, the quantity of bacteria had a significant reduction in the Ce6+ group compared with the L, NL, and NC groups, but the difference between the Ce6+ group and the Ce6 group had no statistical significance. On the 35th day, significant differences were found between the Ce6+ group and the other groups, while this inhibitory effect persisted until rats were killed on the 41st day.

**FIG 6:**
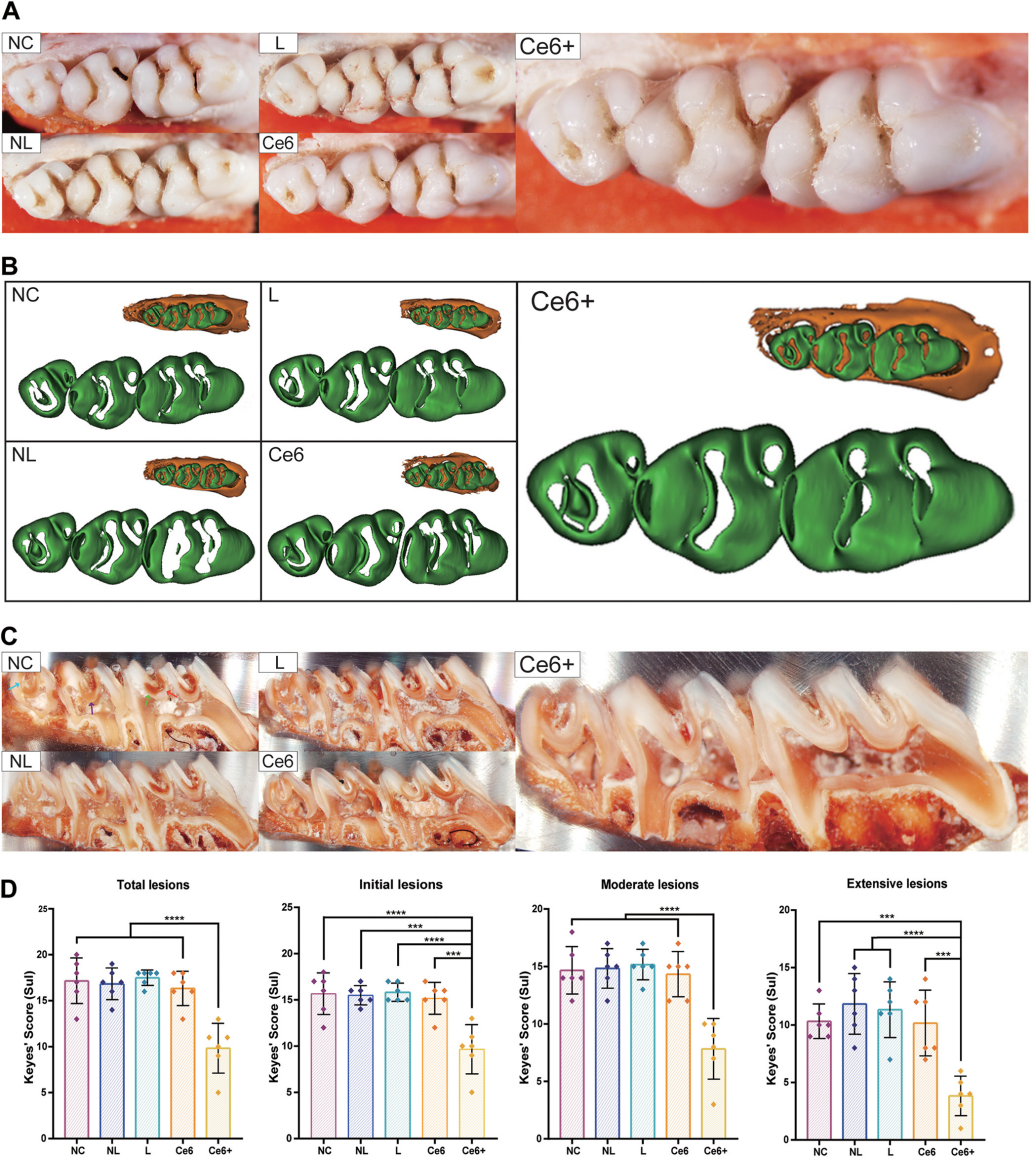
Anticaries effects of Ce6-PDT combined with HSP inhibitors *in vivo*. (A) Photographs of occlusal surface show caries lesions in pits and fissures. (B) Reconstructed images after micro-CT scanning show the enamel loss of occlusal surfaces. (C) Representative photographs of sulcus lesions stained by murexide. Blue arrows point to the lesion penetrating the enamel only. The red arrow indicates a lesion penetrating within one-fourth of the dentin (slight dentinal). The green arrow indicates a lesion penetrating one-fourth to three-fourths of the dentin (moderate dentinal). The purple arrow indicates a lesion penetrating beyond three-fourths of the dentin (extensive dentinal). (D) Keyes’ score of total lesions, initial lesions (involving within slight dentinal), moderate lesions (involving within moderate dentinal), and extensive lesions (involving within extensive dentinal). The results are shown as mean ± SD (*n* = 6); ***, *P < *0.001; ****, *P < *0.0001.

Next, anticaries properties were further observed and analyzed when the caries model was established successfully in the NC group. There was almost no pigmentation in pits and fissures of molars belonging to the Ce6+ group, while mild or severe pigmentation could be observed in other groups (Fig. 6A). At the same time, enamel loss in the Ce6+ group was visually less than all of the other groups (Fig. 6B). Additionally, caries lesions were stained in murexide (Fig. 6C) for Keyes’ score to evaluate the progression of caries clearly. It was confirmed that scores of total, initial, moderate, and extensive lesions were always lower in the Ce6+ group than in the other groups, and no significant difference was found among other groups (Fig. 6D).

**FIG 7:**
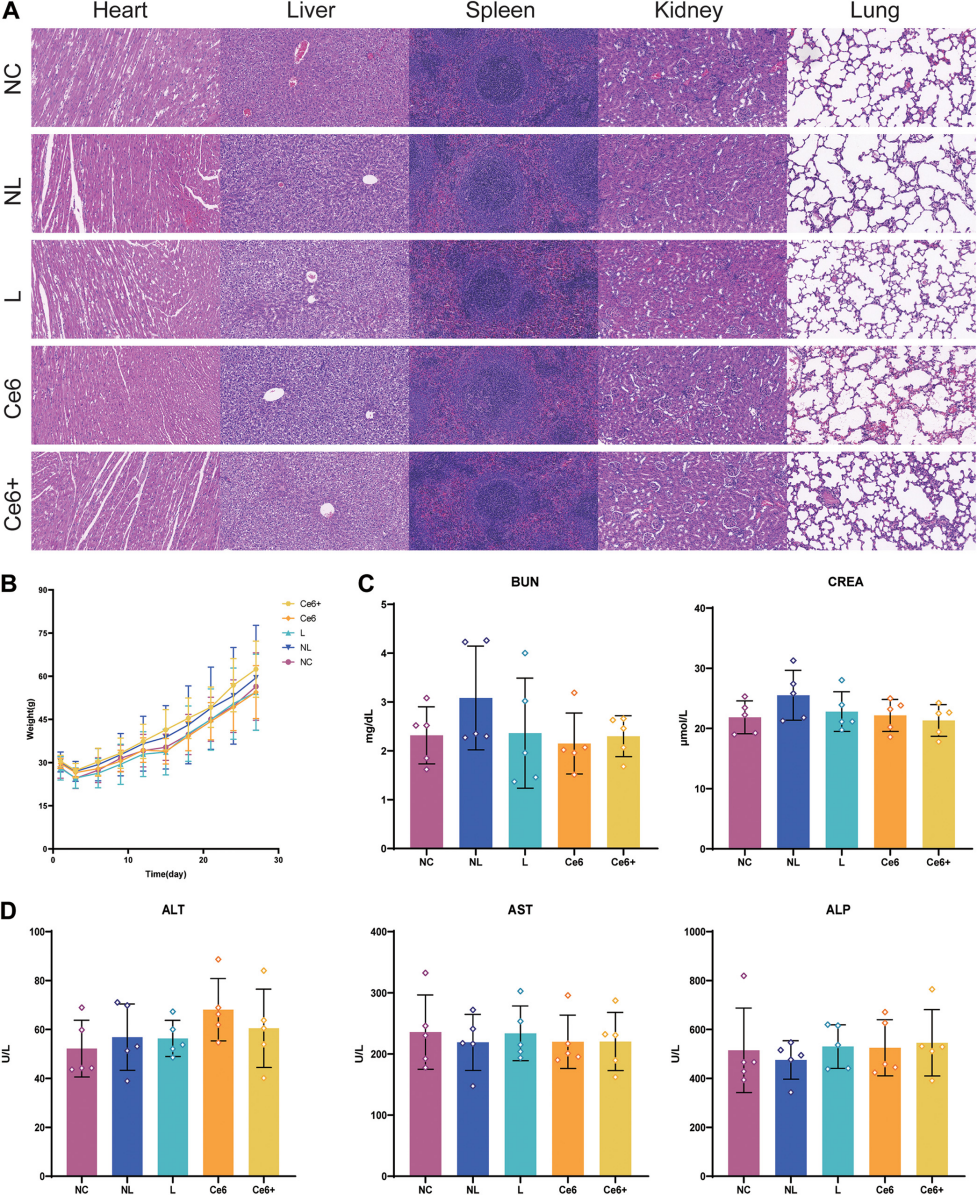
Biological safety of Ce6-PDT combined with HSP inhibitors *in vivo*. (A) Hematoxylin and eosin staining for heart, liver, spleen, lung, and kidney of rats. (B) Weight record of rats during the entire experiment. (C and D) Blood biochemistry analysis. Blood urea nitrogen (BUN) and creatinine (CREA) indicate renal function (C). Alanine aminotransferase (ALT), aspartate aminotransferase (AST), and alkaline phosphatase (ALP) indicate hepatic function (D). The results are shown as mean ± SD (*n* = 5).

Moreover, we evaluated the biological safety of these treatments. Weights of rats had similar growing trends during the whole experiment in each group (Fig. 7B), while the tissue structure and cell morphology of organs (Fig. 7A) and blood constituents (Fig. 7C and D) showed no obvious differences between these groups.

## DISCUSSION

In recent years, PDT has exhibited outstanding performance in the fields of antitumor, antimicroorganism, anti-inflammation, and immunoregulation (36). It is well-established that synergistic use of HSP inhibitors and PDT is more effective in antitumor and anti-inflammation fields (37–39), but it is still rarely discussed as an antibacterial strategy in the field of caries prevention. Our objective in the present study was to evaluate the synergistic inhibitory effects of PDT and HSP inhibitors on cariogenic bacteria *in vitro* and *in vivo* to find a new strategy for preventing caries. This was achieved using CV staining, live/dead bacterial staining, FISH, and FE-SEM assays as well as developing an *in vivo* caries model.

In the current study, the combination of Ce6-PDT and HSP inhibitors showed improved inhibition toward planktonic S. mutans and *S. sobrinus* compared to Ce6-PDT alone and lead to less bacterial residues. However, the role of the combined application in S. sanguinis was not as effective as observed in S. mutans and *S. sobrinus*. Considering that the upregulation of *grol* and *dnak* genes, which was fairly low in S. sanguinis treated with Ce6-PDT, differed from other tested bacteria, we speculated that there may be other essential HSPs or principal mechanisms for S. sanguinis to withstand environmental stresses. At present, S. sanguinis has been considered a controversial bacterium in terms of its different roles in health or disease conditions. Some researchers reported that S. sanguinis virulence factors correlate to bacterial endocarditis (40, 41). In periodontal diseases, S. sanguinis is beneficial for the survival of Fusobacterium nucleatum, and it can protect Aggregatibacter actinomycetemcomitans from the host inflammatory response (42, 43). On the other hand, S. sanguinis is considered one of the most prolific inhabitants on the tooth surface, which plays a dominant role in the process of biofilm development (44). S. sanguinis also has an antagonistic effect against S. mutans (45) and has an inverse association with caries (46). A previous study indicated that the proportion of Streptococcus sanguinis is lower in caries-active individuals than in caries-inactive individuals (10% versus 36%) (10). Taking these findings into consideration, the inconspicuous effect of Ce6-PDT with HSP inhibitors in S. sanguinis is acceptable. We did not conduct further experiments for single S. sanguinis strains in subsequent assays and regarded it as a pioneer colonizer in establishing multispecies microbiofilms.

Before the exploration in multispecies biofilm, our experimental strategy was performed in monomicrobial biofilms of S. mutans and *S. sobrinus*. According to our data, Ce6-PDT with HSP inhibitors exerts remarkable enhancement in inhibiting the formation of monomicrobial biofilm and also has the capacity to suppress the vitality of bacteria in the biofilm. A similar finding was shown in the multispecies biofilm, which is more close to the condition *in vivo*. Briefly, the biomass of multispecies biofilms was remarkably lessened, especially under short-term treatment. Furthermore, amounts of three Streptococcus strains in short-term biofilms were obviously decreased, while more severe destruction of biofilm structure and less cell aggregation were observed in long-term biofilms. As such, Ce6-PDT with HSP inhibitors has a better suppressive effect on the formation of cariogenic biofilms than Ce6-PDT alone.

Herein, these results are very important in indicating the satisfactory effect of the synergistic application of HSP inhibitors and Ce6-PDT *in vitro*; we then focused on an *in vivo* caries model established in rat. On the 35th and 41st days, HSP inhibitors improved the effect of Ce6-PDT on decreasing the quantity of streptococci colonized on the tooth surface. Consistently, the suppression of carious occurrence on occlusal surfaces and the reduction of enamel loss were also substantially enhanced when Ce6-PDT was combined with inhibitors. To our surprise, the extent and severity of caries lesions could not be decreased by Ce6-PDT but were noticeably suppressed by synergistic therapy, which suggests that combining HSP inhibitors with Ce6-PDT has a strong potential to impede the progression of caries in clinical practice. Meanwhile, our experimental strategy did not show any toxicity toward rats. It can be concluded that the synergistic use of Ce6-PDT and HSP inhibitors exerts a superb capacity *in vivo* to diminish oral streptococci and reduce the severity of caries lesion while also having satisfactory biological safety.

PDT, which rarely induces bacterial resistance (47), has produced satisfying effects on several microorganisms related to infectious diseases. Recently, several researchers reported great antibacterial effects of PDT on pathogens of keratitis and periodontitis (48, 49); in the context of caries, PDT also showed effective inhibition toward cariogenic S. mutans and microcosms derived from dentin carious lesions (50, 51). Additionally, Ce6 is an extensively studied PS with plenty of advantages (52), and Ce6-PDT has already been demonstrated to have remarkable antibacterial effects against Streptococcus pneumoniae, Moraxella catarrhalis, and several cariogenic streptococci (53–56). However, Ce6 still has a major disadvantage because it is poorly soluble in water (57), which leads to a lower concentration than desired, potentially limiting its practical application. At the same time, PDT also has an unsolved problem where it may promote the production of HSPs (23), which can protect cells from environmental stresses through repairing or cleaving unfolded proteins (32). In Streptococcus species, the major HSP families who play these protective roles are the GroEL family and DnaK family (31, 32), and they were also validated to be upregulated under Ce6-PDT treatment in our present research (Fig. 1C to E).

Based on the evidence mentioned above, it may be feasible to use a combination of HSP inhibitors with PDT to offset these disadvantages to improve the actual antibacterial effects. St. Denis et al. (23) studied the combination of PDT and HSP 70 inhibitor toward Escherichia coli and Enterococcus faecalis, but the synergistic effect was inconspicuous. Conversely, we gained positive results via incorporating HSP 60 inhibitor and HSP 70 inhibitor into Ce6-PDT and validated PDT with Ce6 at a low dose, which can achieve outstanding antibacterial and antibiofilm effects in the presence of HSP inhibitors, which can remedy the disadvantages of Ce6-PDT. However, our study still has an inadequacy that we used fixed total energy of irradiation, which is a moderate dose among existing studies (58, 59); therefore, further research is necessary to detect whether there is a more appropriate irradiation condition for this therapy. Although a promising effect on the reduction of residual bacteria was observed on agar plates, more specific assays are needed for further evidence. Also of note is that far more experiments are necessary for exploring whether this therapy will have an effect on Gram-negative bacteria and lead to an imbalance of oral microorganisms in a subsequent investigation.

In conclusion, all of our results demonstrate that the combined use of Ce6-PDT and HSP inhibitors has an excellent capacity for inhibiting bacterial proliferation and biofilm formation of three dominant cariogenic bacteria. Moreover, Ce6-PDT and HSP inhibitors could concurrently inhibit the progression of caries and reduce the severity of carious lesion *in vivo*. The current conclusion can thus help in designing new therapeutic methods for the treatment and prevention of caries in the future.

## MATERIALS AND METHODS

### Strains and culture conditions.

Three Streptococcus strains were used in this research: S. mutans (UA159; ATCC, 700610), *S. sobrinus* (ATCC, 27352), and S. sanguinis (SK36; ATCC, BAA-1455D-5). Bacteria were grown in brain heart infusion (BHI) broth (Bacton, Dickinson and Company, USA) under microaerophilic conditions (5% CO_2_, 1% O_2_, 37°C) overnight. For uniformity of all experiments, bacteria suspensions were resuspended to an optical density (OD) of 0.8 at 600 nm (10^9^ CFU/mL) before use.

### Photosensitizer, inhibitors, and light source.

All of the stock solutions (Ce6 [J&K Scientific, Ltd.], apoptozole [MedChemExpress, USA], and nonactin [MKbio, China]) were prepared in dimethyl sulfoxide (DMSO; Biofroxx, Germany) and stored at −80°C in the dark. A laser (LWRPD-1.5F, Laserwave Co., Ltd., China) with red light (660 nm) was chosen as the light source.

### Lethal dose of Ce6-PDT.

Overnight bacterial suspensions were diluted to 2 × 10^7^ CFU/mL. Next, aliquots of 150 μL of bacterial dilutions were poured into 24-well plates. For obtaining the final concentration (800 to 25 nM), the stock solution of Ce6 was diluted 2-fold stepwise and mixed with the dilution in equal volume. Samples were then irradiated with red light (0.5 W/cm^2^, 5 min) after incubating for 1 h in the dark under microaerophilic conditions. Following irradiation and another 15-min incubation in the dark, samples were serially diluted 10-fold, and 10 μL of dilution was spread evenly on BHI agar plates. Plates were incubated under microaerophilic conditions for 48 h, and colonies were counted. The results were converted into log values. At the same time, we set a normal control group, dark control group (without light), and light control group (without Ce6). In this research, the lethal dose (LD) was defined as the drug concentration of PDT that resulted in a 3 log_10_ reduction in CFU, according to previous studies (60, 61).

### Expression of *grol* and *dnak* genes after Ce6-PDT.

Ce6 solution was mixed with the bacterial suspension (2 × 10^7^ CFU/mL) in 24-well plates, and the plates were incubated for 1 h. The final concentrations of Ce6 were 25, 25, and 50 nM for S. mutans, *S. sobrinus*, and S. sanguinis, respectively. Wells were then irradiated with 0.5 W/cm^2^ red light for 5 min and incubated overnight. For quantitative real-time PCR (qRT-PCR) analysis, the suspensions were collected to extract bacterial total RNA with RNAiso reagent (TaKaRa Biotech, China) following the manufacturer’s instructions. RNA from all samples was converted to cDNA with HiScript II Q RT SuperMix (Vazyme Biotech, China) when the purities and concentrations were determined by Nanodrop 2000 (Thermo Fisher, USA). Then, qRT-PCR was conducted using 1,000 ng of cDNA template, 2× ChamQ SYBR qPCR master mix (Vazyme Biotech, China), and primers (Table 1). Reaction conditions were set for 30 s at 95°C, followed by 40 cycles of 95°C for 10 s and 60°C for 30 s. Melting curves were constructed in the build-in program of Bio-Rad CFX96 (Bio-Rad Laboratories, USA) when the reaction was complete. Primers used in this experiment were designed by Sangon Biotech company (Sangon Biotech, China).

**TABLE 1:** Specific primers used in the qRT-PCR assay

Primer	Sequence	Product size (bp)
S. mutans *grol*	Forward 5′-CAGGTGGTGGAACGGCACTTATC-3′	125
	Reverse 5′-AGCAATTTGACGGACAGGCTCTTC-3′	
S. mutans *dnak*	Forward 5′-AACTCCTGTTCGTCAAGCCCTTTC-3′	87
	Reverse 5′-ATACGTGTTGAACCGCCGACTAAG-3′	
S. mutans 16S rRNA	Forward 5′-GCGTGGGTAGCGAACAGGATTAG-3′	117
	Reverse 5′-AGGCGGAGTGCTTATTGCGTTAG-3′	
*S. sobrinus grol*	Forward 5′-GTAACATCGTCCTTCGTGCTCTGG-3′	134
	Reverse 5′-ACTACCATCGGCAGCATTGAAACC-3′	
*S. sobrinus dnak*	Forward 5′-ACTCAGCAGTCGCAGTTCTTGAAG-3′	142
	Reverse 5′-TTGGTTACCGCTTGACGCTTGG-3′	
*S. sobrinus* 16S rRNA	Forward 5′-GCAACGATACATAGCCGACCTGAG-3′	107
	Reverse 5′-TGCGTCCATTGCCGAAGATTCC-3′	
S. sanguinis *grol*	Forward 5′-TGGAAGAGCCTGTTCGTCAAATCG-3′	115
	Reverse 5′-CATTCGCCAGTCGCAGCATTAAAG-3′	
S. sanguinis *dnak*	Forward 5′-AACAATGGGTGGCGTCTTCACTAAG-3′	95
	Reverse 5′-GTTGGTTGTCCGCTGCTGTAGAG-3′	
S. sanguinis 16S rRNA	Forward 5′-GCTCACCAAGGCGACGATACATAG-3′	113
	Reverse 5′-CCCATTGCCGAAGATTCCCTACTG-3′	

### Synergistic effect of Ce6-PDT and HSP inhibitors on planktonic bacteria.

Bacterial cultures were diluted to 3 × 10^7^ CFU/mL and added in 24-well plates. Afterward, Ce6 solution was serially diluted 2-fold (150 to 37.5 nM) and mixed with cultures in equal volumes. Apoptozole and nonactin solutions were added into the wells at final concentrations of 40 μM and 4 μM, respectively, and incubated for 1 h. Wells were then illuminated with red light (0.5 W/cm^2^, 5 min) and incubated for another 15 min. Each sample was diluted 1,000-fold, and 10 μL of dilution was inoculated evenly on BHI agar plates, followed by 48 h of culture and colony counting. In this part, we set normal controls, dark controls (without light), light controls (without Ce6 and inhibitor), and lighting with inhibitor controls (without Ce6).

### Formation of short- and long-term biofilm.

In the short-term experiment, two biofilm models (monomicrobial biofilm and multispecies biofilm) were constructed, and BHI broth containing 1% sucrose served as the culture medium. To construct the monomicrobial biofilm, three bacterial suspensions were diluted to 3 × 10^7^ CFU/mL and placed, respectively, in different 24-well plates. Ce6, apoptozole, and nonactin solutions were added into the wells at final concentrations of 100 to 25 nM, 40 μM, and 4 μM, respectively, followed by a 4-h incubation. The supernatant was then removed, and wells were directly exposed to red light (0.5 W/cm^2^, 5 min). Afterward, fresh BHI broth was poured into wells, and the plates were cultured under microaerophilic conditions for 24 h. For constructing multispecies biofilms, three bacterial suspensions were diluted to 3 × 10^7^ CFU/mL and mixed in equal volume. Samples were then prepared as described above.

In the long-term experiment, only the multispecies biofilm model was constructed. Bacterial suspensions were cocultured with Ce6 solution, apoptozole, and nonactin solutions at the same concentrations mentioned previously for 4 h. Supernatant was then removed, and wells were exposed to red light (0.5 W/cm^2^) for 5 min. Afterward, fresh BHI broth was added, and the plates were cultured for 20 h. This treatment was repeated until multispecies biofilms had been cultured for 72 h.

In this part, we set normal controls, dark controls (without light), and light controls (without Ce6 and inhibitor).

### Synergistic effect of Ce6-PDT and HSP inhibitors on biofilm formation.

Total biofilm biomass was detected by the crystal violet assay. Briefly, constructed biofilms were stained with 0.1% crystal violet solution for 15 min. The dye was then extracted by 30 to 40% acetic acid, and OD values of the extract were read at 562 nm. Additionally, monomicrobial biofilms were stained with a bacterial viability kit (LIVE/DEAD BacLight, Invitrogen, USA), following the manufacturer’s protocol, and visualized with confocal laser scanning microscopy (CLSM; Leica SP8, Germany). Fluorescence *in situ* hybridization (FISH) was performed on short-term multispecies biofilms according to the manufacturer’s protocol. Bacterial strains were labeled with specific oligonucleotide probes (Sangon Biotech Co., Ltd., China) and scanned using CLSM. The excitation/emission wavelengths were 496/519 nm for Alexa Fluor 488, 346/448 nm for aminomethyl coumarin, and 589/615 nm for Texas Red. Additionally, long-term multispecies biofilms were fixed with 4% glutaraldehyde overnight, followed by gradient dehydration (75%, 80%, 90%, 95%, and 100% ethanol). Critical point drying and gold coating were performed before samples were observed by field-emission scanning electron microscopy (FE-SEM; Sigma, Zeiss, Germany).

### *In vivo* experiment.

The animal experiment protocol was approved by the Animal Ethics Committee for Experimental Research of Wuhan University (permission number S07922040H) and was conducted following the committee’s guidelines. The caries model was established following our previous study with some modifications (55). Briefly, 2-week-old Sprague-Dawley rats were fed with antibiotic water (200 μg/mL benzylpenicillin and 1,500 μg/mL streptomycin sulfate) and antibiotic feed (1 g/kg) for 3 days. We then swabbed plaques from occlusal surfaces using a cotton swab and kept the swabs in phosphate-buffered saline (PBS; HyClone, USA) (62). Portions of samples were spread evenly on Mitis Salivarius agar (MSA) plates (containing 0.2 U/mL bacitracin and 0.01 mg/mL potassium tellurite) after sonic oscillation (63, 64). Colony counting was then performed to detect the effect of the antibiotic diet. Subsequently, rats were fed with a cariogenic diet 2000 (Jiangsu Xietong Pharmaceutical Bioengineering Co., Ltd., China) until the end. Following bacterial inoculation, three bacterial suspensions were mixed to a final concentration of 1 × 10^8^ CFU/mL and inoculated on the maxillary molars of rats. After a 3-day inoculation, plaque samples were collected and counted, while rats that were inoculated successfully were distributed into 5 groups: Ce6 + inhibitor, Ce6, light only, Ce6 + inhibitor (without light), and normal control. PDT began on the 21st day, and the concentrations of Ce6, apoptozole, and nonactin were 500 nM, 200 μM, and 20 μM, respectively. Maxillary molars were treated with drug and irradiated with red light (0.5 W/cm2, 3 min) under inhalation anesthesia. Irradiation was performed for 3 days, and irradiation was then administered every 2 days until the 41st day. In the irradiation cycle, plaque samples were collected and cultured in the same way previously mentioned on the 29th day, 35th day, and 41st day.

All rats were killed by CO_2_ asphyxiation on the 41st day, and their maxilla were collected to be photographed by a stereoscopic microscope. The level of caries was assessed by microcomputed tomography (micro-CT) and the Keyes’ scoring method after staining using murexide (60 mg/mL) (65). Additionally, the blood, heart, liver, spleen, lung, and kidney were collected to evaluate biological safety via blood biochemistry analysis and hematoxylin and eosin staining.

### Statistical analyses.

All of the data were obtained from at least three independent experiments and are presented as means ± standard deviation (SD). Analyses were performed using Prism 8.0 (GraphPad Software, USA). To determine statistical significance, a Student’s *t* test was chosen for the comparison between two groups, and a one-way analysis of variance (ANOVA) was used to compare three or more groups. A *P *value of <0.05 was considered statistically significant.
